# Antimicrobial Resistance in Pediatric UTIs with Congenital Urogenital Anomalies: An 11-Year Saudi Retrospective Study

**DOI:** 10.3390/antibiotics15050506

**Published:** 2026-05-18

**Authors:** Fuad Alanazi, Basmah M. Almaarik

**Affiliations:** Department of Clinical Laboratory Sciences, College of Applied Medical Sciences, King Saud University, Riyadh 12372, Saudi Arabia; balmaarik@ksu.edu.sa

**Keywords:** urinary tract infection, congenital urogenital anomalies, antimicrobial resistance, pediatrics, multidrug resistance, *Escherichia coli*

## Abstract

Background/Objectives: Children with congenital urogenital anomalies (CUA) face increased risk of urinary tract infections (UTIs) and may harbor resistant organisms due to recurrent infections and antibiotic exposure. This study characterized the distribution of uropathogens and antimicrobial resistance patterns at a tertiary center in Saudi Arabia. Methods: This retrospective cohort study included pediatric patients (<18 years) with documented congenital urogenital anomalies and positive urine cultures at King Khalid University Hospital, Riyadh (2015–2025). Susceptibility interpretations (S/I/R) were extracted from the hospital laboratory information system; multidrug resistance (MDR) was defined using organism-specific Magiorakos criteria. Results: A total of 168 patients (72.0% male; mean age 4.1 ± 4.5 years) contributed 411 UTI episodes. Among 403 mono-organism episodes (after excluding eight polymicrobial cultures), *Escherichia coli* predominated (150/403, 37.2%), followed by *Klebsiella pneumoniae* (96/403, 23.8%) and *Pseudomonas aeruginosa* (33/403, 8.2%). High resistance was observed for ampicillin (83.6%), trimethoprim-sulfamethoxazole (54.2%), and cephalosporins (cefazolin 62.8%, cefotaxime 35.6%). Carbapenems (2.9%) and aminoglycosides (9.2%) retained >90% susceptibility. Overall MDR was 35.5%, highest among *Klebsiella oxytoca* (57.1%) and *Escherichia coli* (47.6%). Recurrent infections showed numerically higher unadjusted resistance than single episodes. Conclusions: Pediatric patients with congenital urogenital anomalies showed high first-line antibiotic resistance. Carbapenems and aminoglycosides retained predominantly susceptible in vitro profiles in this cohort and may inform empiric considerations alongside ongoing local susceptibility surveillance for this high-risk population.

## 1. Introduction

Congenital urogenital anomalies represent a diverse group of structural malformations that collectively affect approximately three to six per 1000 live births and, particularly those affecting the kidney and urinary tract, constitute a leading cause of chronic kidney disease in children [1,2,3]. This spectrum of abnormalities includes vesicoureteral reflux (VUR), posterior urethral valves (PUVs), ureteropelvic junction obstruction, hydronephrosis, multicystic dysplastic kidney, and various duplications of the collecting system. Children with these anomalies face a markedly elevated risk of urinary tract infections due to impaired urinary flow dynamics, urinary stasis, and, in many cases, the need for intermittent catheterization or indwelling urinary devices [4,5,6,7].

Urinary tract infections in children with structural urinary abnormalities carry significant clinical consequences beyond the immediate morbidity of the acute infection. Recurrent or poorly treated UTIs in this population may lead to renal scarring, progressive nephropathy, hypertension, and ultimately end-stage renal disease [1,3,8]. The management of these infections is therefore critical not only for symptom resolution but also for the preservation of long-term renal function.

Antimicrobial resistance represents one of the most pressing global health challenges of the 21st century, with particularly concerning implications for pediatric populations where treatment options are inherently more limited due to age-related contraindications and a narrower therapeutic index [9,10]. Children with congenital urogenital anomalies may be at heightened risk for harboring resistant organisms due to several interrelated factors: recurrent UTI episodes necessitating repeated therapeutic antibiotic courses; prolonged prophylactic antimicrobial regimens commonly prescribed to prevent recurrence; frequent urinary catheterization that may introduce resistant flora; and increased healthcare exposure from surgical interventions, imaging procedures, and routine follow-up visits [11,12,13].

The selection of appropriate empiric antibiotic therapy for UTIs depends on the knowledge of the local epidemiology of uropathogens and their susceptibility profiles. However, most existing clinical practice guidelines for pediatric UTI management are derived from studies of healthy children with anatomically normal urinary tracts, and the recommendations may not accurately reflect the uropathogen profile encountered in children with structural abnormalities [14,15,16,17]. The majority of published antimicrobial resistance surveillance data originate from North American and European populations, with relatively limited representation from the Middle East and Gulf Cooperation Council (GCC) regions, where resistance patterns may differ considerably due to variations in antibiotic prescribing practices, infection control measures, and population demographics [18,19].

Saudi Arabia, as the largest country in the Arabian Peninsula with a population exceeding 35 million, has experienced rapid modernization of its healthcare infrastructure over the past several decades. Tertiary referral centers now provide full-spectrum management of complex pediatric urological conditions, yet local antimicrobial resistance data specific to this high-risk pediatric subpopulation remain sparse. Understanding the distribution of organisms and resistance patterns in pediatric patients with congenital urogenital anomalies is essential for optimizing empiric antibiotic selection, implementing effective antimicrobial stewardship programs, and ultimately improving clinical outcomes.

Based on regional pediatric UTI and antimicrobial resistance literature from the GCC and Saudi Arabia [18,19,20,21] and on the organism distributions reported in international CAKUT cohorts [22,23], we hypothesized a priori that the microbiology and resistance profile of CAKUT-associated UTIs at a Saudi tertiary referral center might differ from international reports. Putative drivers include regional differences in antimicrobial prescribing pressure and resistance ecology; tertiary referral case mix that concentrates children with complex congenital anomalies and repeated healthcare exposure; and anticipated institutional variation in surgical timing, urinary instrumentation, and antibiotic prophylaxis practices. Direct comparative data on pediatric urology protocols across Saudi, European, and East Asian CAKUT cohorts remain limited; therefore, we framed this study as a regionally anchored characterization rather than a global generalization, providing a benchmark against which cohorts from other healthcare system settings can be compared.

We therefore conducted an 11-year retrospective study at a major tertiary care academic medical center in Riyadh, Saudi Arabia, with the following objectives: (1) to characterize the distribution of causative organisms in pediatric patients with congenital urogenital anomalies presenting with culture-proven UTIs; (2) to assess antimicrobial resistance patterns across seven major antibiotic classes; (3) to determine the prevalence of multidrug resistance; and (4) to compare resistance patterns between patients with single versus recurrent infection episodes.

## 2. Results

### 2.1. Patient Characteristics

A total of 168 patients met the inclusion criteria and were included in the analysis, contributing 411 UTI episodes over the 11-year study period (Table 1). The cohort was predominantly male, with 121 patients (72.0%) being boys and 47 (28.0%) being girls, reflecting the higher overall prevalence of congenital urogenital anomalies in the male population. The median age at the first documented UTI episode was 2.0 years (IQR 0–7), and the mean age was 4.1 ± 4.5 years (range 0–17), reflecting the right-skewed distribution typical of pediatric CAKUT populations, where the majority of cases present in infancy and early childhood.

When stratified by age group, most patients were toddlers aged 1 to 5 years (71/168, 42.3%), followed by infants younger than 1 year (47/168, 28.0%), children aged 6 to 12 years (41/168, 24.4%), and adolescents aged 13 to 17 years (9/168, 5.4%). Regarding infection recurrence, 89 patients (53.0%) experienced recurrent infections defined as two or more UTI episodes during the study period, while 79 patients (47.0%) had only a single documented infection episode. The number of UTI episodes per patient ranged from one to 14, with 89 patients experiencing recurrent infections, which accounted for 332 of the 411 total episodes.

The 411 culture-positive UTI episodes comprise 403 mono-organism episodes and eight polymicrobial episodes; polymicrobial episodes were retained in the cohort denominator but excluded from organism-attributed resistance and MDR analyses. Abbreviations: SD, standard deviation; y, years; UTI, urinary tract infection.

### 2.2. Distribution of Causative Organisms

Among the 403 mono-organism episodes retained for organism-attributed analyses (after the exclusion of eight polymicrobial cultures, see Section 4.2), *Escherichia coli* was the predominant uropathogen, accounting for 150/403 isolates (37.2%) (Supplementary Table S1, Supplementary Figure S1). *Klebsiella pneumoniae* was the second most common organism with 96/403 isolates (23.8%), followed by *Pseudomonas aeruginosa* (33/403, 8.2%), *Enterococcus faecalis* (22/403, 5.5%), and *Enterobacter cloacae* (15/403, 3.7%). Other organisms identified included *Proteus mirabilis* (12/403, 3.0%), *Acinetobacter baumannii* (11/403, 2.7%), *Klebsiella oxytoca* (7/403, 1.7%), *Serratia marcescens* (6/403, 1.5%), and *Citrobacter freundii* (5/403, 1.2%). Gram-negative organisms accounted for 357 of 403 mono-organism episodes (88.6%).

The distribution of organisms varied across age groups (Supplementary Table S2). The proportion of *E. coli* infections was the highest among infants (42/103, 40.8%) and declined with increasing age, reaching 30.4% (7/23) among adolescents. Conversely, *K. pneumoniae* showed the opposite pattern, with the highest proportion observed in adolescents (10/23, 43.5%) compared with infants (25/103, 24.3%). *P. aeruginosa* was most prevalent in the school-age group of 6 to 12 years (13/114, 11.4%).

### 2.3. Antimicrobial Resistance Patterns

**Figure 1:**
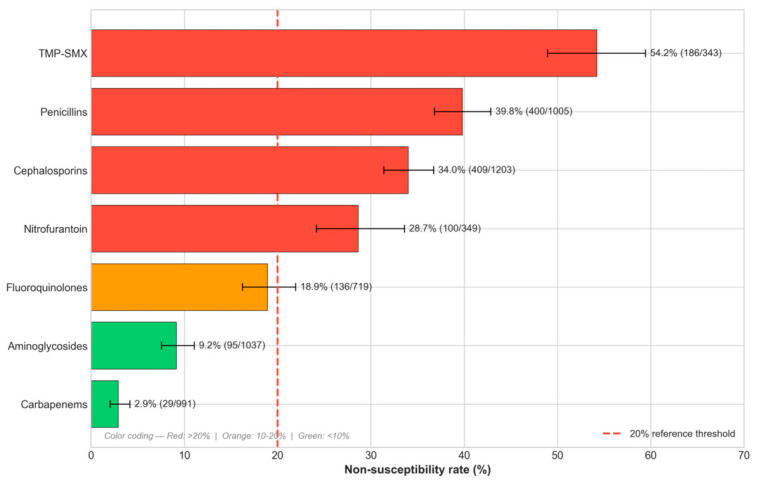
Antimicrobial non-susceptibility rates (I + R per Magiorakos consensus [25]) of uropathogens by antibiotic class. Error bars represent 95% confidence intervals calculated using the Wilson score method [26].

Susceptibility data were available for 410 of the 411 culture-positive episodes (99.8%); one isolate had only non-interpretable results reported. Throughout the manuscript, denominators are used as follows: 411 = full cohort of culture-positive episodes; 410 = subset with interpretable susceptibility data (used for cohort-level recurrence comparison, Section 2.7); 403 = mono-organism episodes (excluding eight polymicrobial cultures used for organism-attributed resistance, Section 2.2 and Section 2.4); 369 = MDR-evaluable subset of monomicrobial isolates tested against ≥3 Magiorakos categories (used for MDR analyses, Section 2.6). All class-level proportions and the MDR endpoint follow the I + R non-susceptibility convention per the Magiorakos consensus framework, as defined in Section 4.5 and Section 4.9. Overall antimicrobial resistance rates by antibiotic class are presented in Supplementary Table S3 and Figure 1. Denominators vary across antibiotics because susceptibility panels differed by organism and clinician request; not all agents were tested on every isolate.

The highest non-susceptibility rate was observed for trimethoprim-sulfamethoxazole, with 186 of 343 tested isolates (54.2%; 95% CI: 48.9–59.4%) demonstrating non-susceptibility. This non-susceptibility rate is well above the 20% threshold, beyond which the empiric use of an antimicrobial agent is generally not recommended by international guidelines [24]. The penicillin class had the second highest non-susceptibility rate at 39.8% (400/1005; 95% CI: 36.8–42.9%), driven primarily by ampicillin, which was the single most-resisted agent with 83.6% non-susceptibility across Enterobacteriaceae with available susceptibility data (Supplementary Table S3), followed by cephalosporins at 34.0% (409/1203; 95% CI: 31.4–36.7%) and nitrofurantoin at 28.7% (100/349; 95% CI: 24.2–33.6%). Fluoroquinolone non-susceptibility was observed in 18.9% of tested isolates (136/719; 95% CI: 16.2–21.9%), approaching but not exceeding the 20% threshold.

In contrast, the aminoglycoside class maintained a favorable susceptibility profile with only 9.2% non-susceptibility (95/1037; 95% CI: 7.6–11.1%). Among individual aminoglycosides, amikacin had the lowest non-susceptibility at 0.9% (3/344), compared to tobramycin at 9.8% (32/327) and gentamicin at 16.4% (60/366) (Supplementary Table S5). Carbapenems had the lowest non-susceptibility rate among all classes tested, at 2.9% (29/991; 95% CI: 2.0–4.2%). Meropenem showed the lowest individual non-susceptibility at 0.9% (3/339), followed by imipenem at 2.3% (8/343) and ertapenem at 5.8% (18/309). Piperacillin-tazobactam susceptibility was also favorable at 97.3% (Supplementary Table S5).

### 2.4. Organism-Specific Resistance Patterns

**Figure 2:**
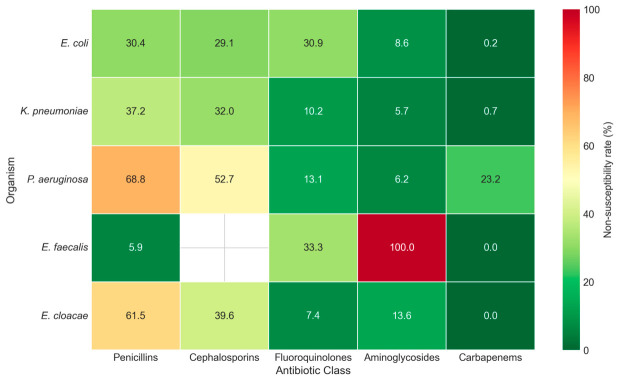
Heatmap of antimicrobial non-susceptibility rates (% I + R per Magiorakos consensus) for the five most common uropathogens across major antibiotic classes. Antibiotic class abbreviations (TMP-SMX, Pen, Nitro, Ceph, FQ, AG, Carb) are as defined in Table 2.

Antimicrobial resistance patterns varied markedly among different bacterial species (Table 2, Figure 2). *P. aeruginosa* showed consistently elevated resistance across multiple antibiotic classes, with resistance rates of 68.8% for penicillins, 52.7% for cephalosporins, and 23.2% for carbapenems. This organism is intrinsically resistant to both nitrofurantoin and trimethoprim-sulfamethoxazole (per CLSI intrinsic resistance guidance [27]); therefore, these agents were excluded from pooled class-level calculations. *P. aeruginosa* showed relatively lower acquired resistance to fluoroquinolones (13.1%) and aminoglycosides (6.2%).

*E. coli*, despite being the most common pathogen, exhibited high TMP-SMX resistance (64.3%) and moderate fluoroquinolone resistance (30.9%). However, *E. coli* isolates maintained excellent susceptibility to carbapenems (0.2% non-susceptibility), nitrofurantoin (5.1% non-susceptibility), and aminoglycosides (8.6% non-susceptibility). *K. pneumoniae* showed a somewhat more favorable resistance profile compared to *E. coli*, with lower TMP-SMX resistance (47.7%) and fluoroquinolone resistance (10.2%), though penicillin (37.2%) and cephalosporin (32.0%) resistance remained substantial.

*Enterobacter cloacae* showed high penicillin resistance (61.5%), consistent with the inducible chromosomal AmpC β-lactamase characteristic of this species; the extended-spectrum cephalosporin resistance observed (39.6%) likely reflects AmpC derepression under β-lactam pressure, with possible contribution from acquired extended-spectrum β-lactamases that were not characterized in this retrospective dataset. TMP-SMX resistance was 46.7%, and no carbapenem-resistant *E. cloacae* isolates were identified (0.0%). *Acinetobacter baumannii* represented a small proportion of the cohort (*n* = 11 isolates), and non-susceptibility rates should therefore be interpreted as preliminary surveillance signals rather than stable estimates; carbapenem non-susceptibility was observed at 23.5%, and all 11 isolates were non-susceptible to nitrofurantoin (100%), consistent with the characteristically poor clinical activity of nitrofurantoin against this species.

### 2.5. Annual Resistance Proportions, 2015–2025

**Figure 3:**
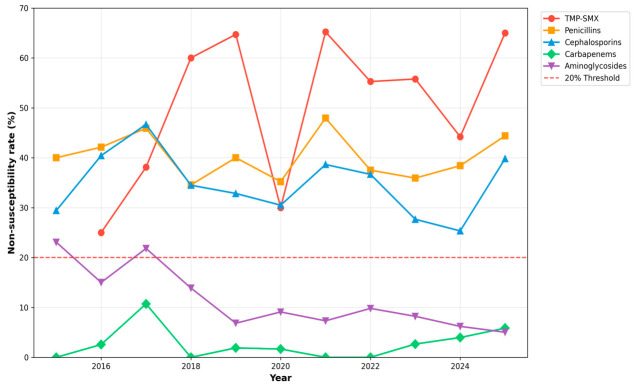
Annual uropathogen antimicrobial non-susceptibility proportions from 2015 to 2025. The horizontal dashed red line indicates the 20% empiric therapy threshold. Annual proportions are presented descriptively as cross-sectional snapshots; year-to-year differences should not be interpreted as evidence of temporal change in true non-susceptibility prevalence, given that the laboratory’s interpretive standard and breakpoint version could not be reconstructed across the study period.

Annual non-susceptibility proportions from 2015 through 2025 are shown descriptively in Figure 3. TMP-SMX and penicillin non-susceptibility consistently exceeded the 20% empiric therapy threshold across all years of observation. Carbapenem and aminoglycoside non-susceptibility remained below 10% in every year. Cephalosporin non-susceptibility varied year-to-year between approximately 25% and 43% with no clear directional pattern. The Cochran–Armitage linear-by-linear trend test showed no statistically significant change in the overall MDR rate across 2015–2025 (Z = 0.36, *p* = 0.72); however, because the laboratory’s interpretive standard (CLSI versus EUCAST) and breakpoint version could not be reconstructed across the study period, this analysis is reported as exploratory only and should not be interpreted as evidence of true temporal stability in resistance. Annual proportions should be regarded as cross-sectional snapshots, particularly noting that the relatively small number of susceptibility tests in some year–antibiotic combinations produces wide year-to-year fluctuations that should not be over-interpreted.

### 2.6. Multidrug Resistance

Among the 369 isolates evaluable for MDR classification (tested against at least three organism-specific Magiorakos categories), 131 (35.5%; 95% CI 30.8–40.5%) met the criteria for multidrug resistance. A sensitivity analysis restricted to the earliest MDR-evaluable episode per patient yielded a near-identical estimate (55/156 patients, 35.3%; 95% CI 28.2–43.0%), indicating that episode-level pooling did not inflate the MDR rate. Table 3 presents per-organism MDR rates for organisms with five or more isolates, which together account for 351 of the 369 evaluable isolates. MDR rates varied by organism. *Klebsiella oxytoca* showed the highest MDR rate at 57.1% (4/7), followed by *Proteus mirabilis* (6/12, 50.0%) and *E. coli* (70/147, 47.6%). *K. pneumoniae* had an MDR rate of 38.3% (36/94), while *Enterobacter cloacae* showed 26.7% (4/15) after excluding intrinsic AmpC-mediated resistance. *P. aeruginosa* had an MDR rate of only 6.1% (2/33) when assessed against the five antipseudomonal-specific Magiorakos categories, indicating that its high overall resistance rates (Table 2) predominantly reflect intrinsic rather than acquired resistance mechanisms.

Among the two most common pathogens, *E. coli* had an MDR rate of 47.6% (70/147 evaluable isolates), and *K. pneumoniae* had an MDR rate of 38.3% (36/94 evaluable isolates). Point estimates for less common organisms with small sample sizes, notably *Klebsiella oxytoca* (57.1%, 4/7) and *Proteus mirabilis* (50.0%, 6/12), should be interpreted cautiously given their wide 95% confidence intervals (25.0–84.2% and 25.4–74.6%, respectively). *Enterococcus faecalis* had a 0% MDR rate (0/16 evaluable isolates), as this organism remained susceptible to ampicillin and vancomycin, and intrinsic resistance to certain classes (cephalosporins and aminoglycosides for synergy) was not counted toward the MDR definition per consensus guidelines.

In multivariable logistic regression adjusted for age group, sex, CAKUT phenotype, and recurrent infection status (clustered on patient), hypospadias showed higher MDR odds in the primary model compared to hydronephrosis (aOR 2.07, 95% CI 1.03–4.14, *p* = 0.040; borderline statistical significance with lower CI near unity), while male sex was independently associated with lower MDR odds compared to female sex (aOR 0.34, 95% CI 0.16–0.73, *p* = 0.006). Age group, PUV, VUR, and recurrent infection status were not independent predictors after adjustment. Full model estimates are presented in Table 4. In a sensitivity analysis restricted to the first isolate per patient (*n* = 156), the MDR rate was 35.3% (55/156), closely matching the episode-level estimate of 35.5% and indicating that the primary analysis was not meaningfully biased by patients contributing multiple episodes. The hypospadias subgroup contributed 37 patients with 70 culture-positive episodes overall (69 MDR-evaluable), of which 28 (40.6%) met MDR criteria. Within the MDR-evaluable cohort (156 patients), 106 (67.9%) had at least one documented surgical procedure recorded in the patient-level dataset, and six (3.8%) had documented stent, catheter, or vesicostomy exposure. In an extended multivariable GEE that additionally adjusted for any documented surgical procedure, stent/catheter/vesicostomy exposure, and total infection episode count (Supplementary Table S6, Model B), the hypospadias point estimate changed minimally (aOR 2.03, 95% CI 0.98–4.23, *p* = 0.058) compared with the primary model (aOR 2.07, 95% CI 1.03–4.14, *p* = 0.040); precision decreased and the 95% confidence interval crossed the null after adding these procedural exposure proxies. In a documented procedure subgroup sensitivity analysis restricted to the 106 patients (252 episodes) with a non-empty surgery field (Supplementary Table S6, Model B-DocSurg), the hypospadias point estimate attenuated further (aOR 1.71, 95% CI 0.80–3.67, *p* = 0.165). This subgroup analysis supports a cautious interpretation of the hypospadias-associated signal but is not a complete-case correction, as we cannot determine from the available data whether empty surgery values indicate missing data or a true absence of a documented procedure. None of the procedural exposure covariates was independently associated with MDR in the extended model (Has_Surgery *p* = 0.52, stent/catheter/vesicostomy *p* = 0.40, total infection episode count *p* = 0.48). The lower MDR odds in males remained stable in the extended model (aOR 0.32, 95% CI 0.15–0.69, *p* = 0.004). Age-stratified MDR proportions and class-level non-susceptibility patterns are reported descriptively in Supplementary Table S7. MDR proportions were broadly similar across age groups (infant 36.4%, toddler 39.1%, child 29.6%, adolescent 33.3%; Wilson 95% confidence intervals overlapping); class-level non-susceptibility did not show systematic age-dependent shifts beyond a higher TMP-SMX non-susceptibility in toddlers (62.4%) compared with adolescents (35.0%) and a numerically higher carbapenem non-susceptibility in adolescents (8.9%) compared with younger age groups (1.6–4.0%). Formal inferential testing across age strata was not performed because the adolescent stratum was small (21 episodes/nine patients in the MDR-evaluable cohort).

### 2.7. Resistance by Infection Recurrence Status

Comparison of antimicrobial resistance rates between patients with single infection episodes (*n* = 79 isolates with susceptibility data) and those with recurrent infections (*n* = 331 isolates with susceptibility data, from 89 patients with ≥2 episodes), totaling 410 episodes at the cohort-level recurrence comparison denominator (411 culture-positive episodes minus one episode with non-interpretable AST; polymicrobial episodes are retained here because recurrence comparison does not require organism attribution) showed numerically higher unadjusted resistance in the recurrent infection group across all antibiotic classes examined, though no formal inferential comparison was performed (Supplementary Table S4). The differences were most pronounced for nitrofurantoin (31.0% vs. 13.9%), fluoroquinolones (21.1% vs. 8.7%), and penicillins (41.6% vs. 30.7%). TMP-SMX resistance was elevated in both groups, but higher in recurrent infections (55.8% vs. 48.7%). Carbapenem resistance, while low in both groups, was also higher in patients with recurrent infections (3.3% vs. 1.5%).

## 3. Discussion

This 11-year retrospective study of 168 pediatric patients with congenital urogenital anomalies and 411 culture-proven UTI episodes at a Saudi tertiary referral center provides organism-level and antimicrobial resistance data for a high-risk population in whom routine community pediatric UTI assumptions may not apply.

A key finding is the high prevalence of non-susceptibility to commonly prescribed first-line agents. TMP-SMX non-susceptibility exceeded 54%, more than double the 20% threshold above which international guidelines recommend against empiric use of an antimicrobial agent [24]. Penicillin-class non-susceptibility approached 40%, and cephalosporin-class non-susceptibility exceeded 33%. These rates are higher than those typically reported in community-acquired pediatric UTIs among otherwise healthy children, where *E. coli* resistance to TMP-SMX generally ranges from 20 to 24% in outpatient pediatric UTI studies [28,29], although community-acquired UTI studies from Saudi Arabia have reported higher rates of 46–57% [30,31,32]. The elevated burden in our cohort may reflect repeated antibiotic exposure among children with congenital urogenital anomalies who experience recurrent infections and often receive prolonged antimicrobial prophylaxis.

The observed organism distribution also differed from that typically reported in community-acquired pediatric UTIs. *E. coli* remained the predominant pathogen at 37.2%, but this proportion is far below the approximately 80% predominance commonly reported in otherwise healthy children [28]. Conversely, *K. pneumoniae* (23.8%) and *P. aeruginosa* (8.2%) were proportionally more common than in community pediatric UTI populations. This shift may reflect selection of more resistant organisms under repeated antibiotic pressure, acquisition of healthcare-associated flora through frequent clinical contact, and altered urinary tract colonization related to structural abnormalities, catheterization, or surgical intervention. The relatively high prevalence of *P. aeruginosa* is clinically relevant given its intrinsic resistance to multiple antibiotic classes [20], but organism-specific Magiorakos MDR analysis showed acquired multidrug resistance in only two of 33 *P. aeruginosa* isolates (6.1%), suggesting that most antipseudomonal options retained activity in this cohort. Within the 403 mono-organism episodes, *K. pneumoniae* was broadly distributed across CAKUT subgroups, whereas *P. aeruginosa* appeared descriptively more frequent in posterior-urethral-valve episodes (12 of 33, 36.4%) than the cohort PUV baseline (123 of 403, 30.5%). Documented surgery exposure was similar across the three predominant uropathogens (*E. coli* 63.3%, *K. pneumoniae* 69.8%, *P. aeruginosa* 66.7%), suggesting that documented surgery alone does not explain the organism shift. Because the dataset lacked a validated community- versus hospital-acquired classification, the non-*E. coli* predominance cannot be assigned to a single procedural or acquisition pathway.

Our findings are broadly consistent with reports from tertiary pediatric centers managing children with urological abnormalities. In a Chinese cohort of children with congenital urogenital anomalies, *E. coli* accounted for approximately 31% of isolates, and ampicillin resistance exceeded 90% [22]. Romanian CAKUT studies reported a similar organism shift, including *E. coli* in 38.8% and *Klebsiella* in 21.2% of 260 UTI episodes, high resistance to ampicillin and cephalosporins, and TMP-SMX resistance of 56.6% for *E. coli* [23,33]. Jafri et al. [34] likewise found *Pseudomonas* to be the dominant pathogen among children with congenital urogenital anomalies, while *E. coli* predominated in the non-anomaly comparison group. An Ethiopian cohort also identified posterior urethral valves as a UTI risk factor among children with congenital urogenital anomalies [35]. These data support the interpretation that CAKUT and related congenital urogenital anomalies are associated with a pathogen distribution distinct from uncomplicated community pediatric UTIs. Regional data point in the same direction: a GCC pediatric UTI review reported TMP-SMX resistance of 36–56% in Saudi Arabia [18], while a Riyadh multicenter pediatric UTI study found *E. coli* predominance of 77.4% in the general pediatric population, far above the 37.2% observed in our anomaly cohort [21]. Recent Lebanese hospital data also reported high MDR rates for *E. coli* and *Klebsiella* in hospitalized children [36]. Direct protocol-level comparison with the Romanian [23,33,37] and Chinese [22] cohorts remains limited because those reports do not consistently describe surgical timing, staged repair practice, catheter or stent dwell time, or prophylaxis policies; our retrospective LIS-based design also did not capture these variables. Between-cohort resistance differences should therefore be interpreted as potentially influenced by unmeasured procedural and prophylactic practice variation, supporting the need for harmonized multicenter CAKUT reporting.

The overall MDR rate of 35.5% reflects a substantial acquired resistance burden in this population. *E. coli* was the leading contributor, with MDR in 70 of 147 evaluable isolates (47.6%). In contrast, *P. aeruginosa* showed MDR in only 2 of 33 isolates (6.1%) when assessed using antipseudomonal-specific Magiorakos categories, indicating that its high crude resistance profile largely reflects intrinsic rather than acquired resistance. Similarly, after excluding intrinsic AmpC-mediated resistance, *Enterobacter cloacae* showed a lower MDR rate (26.7%) than crude multi-class resistance might suggest. The higher resistance observed among recurrent infections is consistent with cumulative antimicrobial exposure as a selection pressure, and aligns with a Romanian recurrent pediatric UTI study that reported MDR prevalence of 48.5% and associations with urinary tract malformations and continuous antibiotic prophylaxis [37]. MDR is treated here as a microbiological endpoint only; its prognostic and management implications require outcome-anchored prospective studies.

The hypospadias-associated MDR signal should be interpreted cautiously. The primary model showed higher MDR odds compared with hydronephrosis-only phenotypes (aOR 2.07, 95% CI 1.03–4.14, *p* = 0.040). The hypospadias analytic category included 37 patients contributing 70 culture-positive episodes overall; among 69 MDR-evaluable episodes, 28 (40.6%) met MDR criteria. In the extended model adjusting for any documented surgical procedure, stent/catheter/vesicostomy exposure, and infection episode count, the estimate was similar but less precise (aOR 2.03, 95% CI 0.98–4.23, *p* = 0.058). A documented procedure subgroup analysis further attenuated the estimate (aOR 1.71, 95% CI 0.80–3.67, *p* = 0.165), whereas a model replacing binary surgery status with the count of distinct documented surgical procedures (Num_Surgeries; range 0–13) yielded a similar estimate for hypospadias (aOR 2.08, 95% CI 1.02–4.25, *p* = 0.045) and no independent association for procedure count itself (aOR 1.04 per additional surgery, 95% CI 0.94–1.16, *p* = 0.425). Excluding three hypospadias/epispadias records requiring clinical classification review also attenuated the association (aOR 1.96, 95% CI 0.90–4.29, *p* = 0.090 in Model A; aOR 1.97, 95% CI 0.87–4.49, *p* = 0.105 when combined with Model C’). These sensitivity analyses support reporting the finding as exploratory rather than confirmed.

The available procedural variables were imperfect structured data proxies. The surgery field captured only the most recent documented procedure type per patient, the stent/catheter/vesicostomy proxy was sparse (6 of 156 MDR-evaluable patients, 3.8%), and the dataset did not capture surgery timing relative to UTI, catheter or stent duration, continuous antibiotic prophylaxis, hospital admissions, length of stay, or prior antibiotic prescription history. The lower MDR odds in males (aOR 0.34, *p* = 0.006) likely reflect sex-linked differences in CAKUT phenotype distribution. The non-significant Cochran–Armitage test for MDR trend across the study period (*p* = 0.72) is reported as exploratory only because laboratory interpretive standards and breakpoint versions could not be reconstructed. Taken together, the hypospadias-associated signal may function as a surrogate marker for cumulative procedural and healthcare exposure, including staged repair, repeated urethral instrumentation, and possible nosocomial flora acquisition, rather than as evidence of an intrinsic anatomic predisposition.

From a clinical management perspective, the in vitro susceptibility data we report may inform institution-level empiric considerations for pediatric patients with congenital urogenital anomalies presenting with suspected UTI in this and comparable tertiary referral settings. Because clinical outcomes—including treatment success, time to clinical resolution, length of stay, recurrence, and complications—were not ascertained in this microbiology records study (this analysis was approved as retrospective use of laboratory data only), the considerations that follow should be interpreted as informed by in vitro non-susceptibility data and not as validated treatment recommendations. First, TMP-SMX appears unsuitable for empiric monotherapy in this tertiary referral setting in light of the resistance proportions reported above. Empiric use of ampicillin monotherapy or amoxicillin-clavulanate is similarly problematic given the penicillin-class non-susceptibility reported above. Although third-generation cephalosporins are commonly recommended for empiric treatment of complicated pediatric UTIs, the cephalosporin-class non-susceptibility in our cohort suggests that these agents may be inadequate as empiric monotherapy in this tertiary referral setting; clinicians in other settings should rely on their local antibiograms.

Aminoglycosides (9.2% non-susceptibility) and carbapenems (2.9% non-susceptibility) retained microbiological activity against uropathogens in this dataset. For seriously ill patients or those with suspected resistant infections, aminoglycosides such as amikacin (0.9% resistance) may be considered as empiric reserve options in this and comparable tertiary referral settings, acknowledging the need for therapeutic drug monitoring and awareness of nephrotoxicity concerns, particularly in patients with underlying renal abnormalities. Piperacillin-tazobactam also showed favorable susceptibility (97.3% in Supplementary Table S5) and may be considered for empiric therapy in appropriate clinical scenarios. Carbapenems should be reserved for severe infections or when resistant organisms are suspected based on prior culture history, as carbapenem-sparing approaches are essential for preserving the efficacy of these last-line agents.

This study has several key strengths. The 11-year study period and the focus on a well-defined population with congenital urogenital anomalies provide more granular resistance data than general pediatric UTI surveillance, which typically pools all uropathogens across diverse populations. The application of organism-specific Magiorakos MDR categories with explicit exclusion of intrinsic resistance provides a more accurate and reproducible MDR estimate than simple multi-class resistance counts. The comprehensive susceptibility data across seven antibiotic classes, spanning an extended observation window from 2015 to 2025, provides broader context than single-year cross-sectional surveys, while we explicitly refrain from interpreting year-to-year differences as longitudinal trends because the laboratory’s interpretive standard and breakpoint version could not be reconstructed across the study period.

Several limitations should be noted. First, susceptibility interpretations were retrieved as S/I/R categories from the hospital laboratory information system; the automated platform, CLSI versus EUCAST standard, and breakpoint edition used across the 11-year period could not be reconstructed. Annual proportions should therefore be read as cross-sectional snapshots, not as evidence of true temporal stability or change. Second, this was a single-center retrospective study at a tertiary referral center, so the cohort is enriched for complex disease and may overestimate resistance compared with community or secondary care settings. Third, microbiological data were not linked to clinical outcomes such as treatment success, length of stay, recurrence, or complications; the empiric therapy considerations above are therefore informed by in vitro non-susceptibility only. Fourth, molecular characterization of resistance mechanisms, including ESBL and carbapenemase testing, was not performed. Fifth, structured admission status data were insufficient to separate community-acquired from healthcare-associated infections, and symptom data were not consistently captured; some culture-positive episodes may therefore represent asymptomatic bacteriuria or colonization, particularly among patients using clean intermittent catheterization or indwelling urinary devices. Finally, although extended models adjusted for available procedural exposure proxies, these variables remained incomplete: the surgery count was approximated from structured surgical fields, the stent/catheter/vesicostomy proxy was sparse, and the dataset lacked surgery timing, catheter or stent duration, prophylaxis regimen, hospitalization burden, and prior antibiotic prescription history. Residual confounding remains possible, which is why the hypospadias-associated MDR signal is reported as exploratory; the classification sensitivity analysis excluding three reviewed hypospadias/epispadias records further supports this cautious interpretation.

Future research should focus on several key areas. Prospective studies linking MDR status and class-level non-susceptibility to clinical outcomes—including treatment failure, length of stay, recurrence, and complications—are needed to determine whether the MDR prevalence reported here translates into proportional clinical impact and to convert antimicrobial susceptibility data into outcome-validated empiric therapy recommendations. Investigation of the relationship between specific prophylaxis regimens and subsequent development of resistance would inform antimicrobial stewardship efforts in this population. Molecular characterization of resistance mechanisms, including ESBL and carbapenemase screening, would provide a more complete picture of the epidemiology of resistance. Finally, multicenter studies across the Middle East region would help establish regional resistance patterns and facilitate the development of regionally appropriate clinical practice guidelines.

## 4. Materials and Methods

### 4.1. Study Design and Setting

This retrospective cohort study was conducted at King Khalid University Hospital (KKUH), a tertiary care academic medical center affiliated with King Saud University in Riyadh, Saudi Arabia. The study protocol was approved by the Institutional Review Board of King Saud University (IRB No. E-25-10178, approved 24 September 2025) with Exempt status and a waiver of informed consent, given the retrospective use of de-identified medical records with no direct patient contact. All methods were performed in accordance with the Declaration of Helsinki. The study covered culture-proven UTI episodes identified in the hospital’s electronic medical record system from 2015 through 2025.

### 4.2. Study Population

We identified all pediatric patients younger than 18 years at the time of their first documented UTI episode who had confirmed congenital urogenital anomalies and at least one positive urine culture between 2015 and 2025. Patients were identified through a systematic review of the hospital information system and microbiology laboratory databases using relevant diagnostic codes and culture records. The cohort spanned a spectrum of congenital urogenital anomalies, including hydronephrosis, posterior urethral valves, vesicoureteral reflux, hypospadias, epispadias, renal dysplasia, and other structural anomalies of the kidney and urinary tract. Patients with isolated undescended testis or other non-urogenital anomalies were excluded.

Inclusion criteria were: (1) age less than 18 years at the time of first UTI episode; (2) documented congenital urogenital anomaly confirmed by imaging studies (renal ultrasonography, voiding cystourethrogram, magnetic resonance urography, or computed tomography urography) or surgical records; and (3) at least one positive urine culture reported as clinically significant growth (single or polymicrobial) by the hospital microbiology laboratory in accordance with its standard clinical reporting thresholds.

Two distinct exclusions were applied. First, an analytic exclusion was applied at the level of organism-attributed analyses: polymicrobial cultures, defined as urine cultures reported in the laboratory information system (LIS) with two or more distinct uropathogens (organism field containing semicolon-separated taxa), were retained in the cohort denominator (i.e., counted as culture-positive UTI episodes) but excluded from organism-attributed resistance and multidrug resistance calculations because susceptibility results could not be reliably attributed to a single pathogen. Polymicrobial growth as defined above was identified in 8 of 411 culture-positive episodes (1.9%) in the cohort. This proportion should be interpreted in light of how the source data were generated: the LIS export used for this study contains only urine cultures that the hospital microbiology laboratory judged clinically significant and on which antimicrobial susceptibility testing was performed (consistent with the clinically actioned UTI cohort definition described in Section 4.4); cultures triaged by the laboratory as contaminated or below the clinically significant growth threshold do not appear in the LIS export and were therefore not part of either the cohort numerator or the polymicrobial exclusion denominator. The 1.9% polymicrobial proportion thus represents the rate of clinically actioned multi-uropathogen cultures within the AST-tested cohort, not the raw rate of mixed flora that may be encountered during urine collection in catheterized children with congenital urogenital anomalies. Second, a study-level exclusion criterion was applied at recruitment: patients with acquired rather than congenital urinary tract abnormalities (such as neurogenic bladder secondary to spinal cord injury or iatrogenic ureteral stricture) were not eligible for the cohort.

### 4.3. Data Collection

Patient data were extracted from the electronic medical record system by trained study personnel using a standardized data collection form. Variables collected included demographic information (age at first infection, sex), type of underlying congenital anomaly, urine culture results including organism identification, and complete antimicrobial susceptibility profiles. Age at the first documented UTI episode was used for demographic calculations and age group stratification.

### 4.4. Episode Definition

Throughout this study, the cohort comprises clinically actioned UTI episodes as defined by routine institutional practice: each included urine culture met the hospital microbiology laboratory’s clinical reporting threshold, excluding contaminant patterns and growth below the institutional clinically significant cutoff, and antimicrobial susceptibility testing was performed on the isolated uropathogen. This indicates that the treating clinical team and microbiology laboratory judged the episode clinically significant and warranting AST-guided management. We did not additionally apply a structured symptom-based filter because such documentation was not consistently captured in the source records; for transparency, we therefore acknowledge that, particularly in patients with intermittent catheterization or indwelling urinary devices, the cohort may include some culture-positive episodes managed as UTIs in which colonization rather than symptomatic infection cannot be retrospectively excluded. With that operational definition in mind, a UTI episode was defined as a positive urine culture meeting the inclusion criteria described above. During upstream data processing, a 14-day grouping window was applied such that positive cultures from the same patient occurring within 14 days of a previous positive culture were treated as part of the same infection episode (only the initial culture was retained for analysis), while positive cultures occurring more than 14 days apart were treated as separate episodes. This window is consistent with episode definition conventions used in prior pediatric UTI surveillance studies and was intended to avoid counting treatment follow-up or persistent cultures as new infections. Recurrent UTI was defined as two or more distinct episodes of infection in the same patient during the study period.

### 4.5. Microbiological Methods

This is a retrospective cohort study based on secondary data extracted from KKUH LIS. Bacterial identification and antimicrobial susceptibility testing (AST) were performed routinely by the KKUH clinical microbiology laboratory as part of standard patient care during the 11-year study period, using the identification and AST methods in clinical use at the institution at the time of each test and in accordance with the laboratory’s standard operating procedures then in force. According to the KKUH clinical microbiology laboratory, routine identification, and AST during this period included MicroScan, VITEK, and Kirby–Bauer disk diffusion, where clinically applicable. Because the study spans 11 years, laboratory platforms, workflows, and interpretive breakpoints may have changed over time. Categorical susceptible/intermediate/resistant (S/I/R) interpretations were assigned by the laboratory and retrieved from the LIS as originally reported. We did not request platform or method documentation from the laboratory at the time of data extraction, and the LIS export available for this study does not contain a per-isolate method or platform field; consequently, the specific identification platform, AST method per individual isolate, interpretive breakpoint guideline (e.g., Clinical and Laboratory Standards Institute [CLSI] M100 versus European Committee on Antimicrobial Susceptibility Testing [EUCAST]), guideline edition, and breakpoint version applied to any individual isolate across the 11-year period could not be reconstructed from the available data records. Because of this limitation, per-year resistance proportions in this study are treated as cross-sectional snapshots rather than as a longitudinal trajectory of resistance, and any year-to-year differences are not interpreted as evidence of temporal change in true non-susceptibility prevalence. Rows reported only as raw MIC values without a categorical S/I/R interpretation were excluded from resistance calculations. For the purposes of resistance calculations in this study, intermediate (I) results were grouped with resistant (R) results as “non-susceptible”, consistent with the Magiorakos international consensus definitions for multidrug resistance, which count non-susceptibility as I + R [25]. We acknowledge that modern CLSI and EUCAST interpretation of “I” may reflect susceptibility at increased drug exposure (the “susceptible-dose-dependent” framework); our estimates are therefore reported as non-susceptibility rather than absolute clinical resistance.

### 4.6. Antibiotic Classification

Antimicrobial agents tested were grouped into seven clinically relevant classes for analysis: trimethoprim-sulfamethoxazole (TMP-SMX); penicillins (including ampicillin, amoxicillin-clavulanate, ampicillin-sulbactam, and piperacillin-tazobactam); cephalosporins (including cefazolin, cefuroxime, cefoxitin, cefotaxime/ceftriaxone, ceftazidime, and cefepime); nitrofurantoin; fluoroquinolones (including ciprofloxacin, levofloxacin, norfloxacin, and moxifloxacin); aminoglycosides (gentamicin, tobramycin, and amikacin); and carbapenems (ertapenem, imipenem, and meropenem). Additional agents tested but not aggregated into these seven classes (e.g., tigecycline, colistin, aztreonam, vancomycin, linezolid, fosfomycin) are reported individually in Supplementary Table S5. This classification scheme reflects both the pharmacological relationships among agents and their typical clinical applications in the empiric and directed treatment of pediatric UTIs.

### 4.7. Definitions

Multidrug resistance (MDR) was defined as acquired non-susceptibility to at least one agent in three or more antimicrobial categories, following the organism-specific category tables proposed by Magiorakos and colleagues. Rather than applying a single classification across all species, antimicrobial categories were assigned per organism group: Enterobacteriaceae (up to 10 categories from our panel), *Pseudomonas* spp. (5 antipseudomonal categories), *Acinetobacter* spp. (7 categories), and *Enterococcus* spp. (3 categories). Intrinsic resistance was excluded per established microbiological principles: chromosomal AmpC β-lactamase-mediated resistance to ampicillin, amoxicillin-clavulanate, first/second-generation cephalosporins, and cephamycins was excluded for *Enterobacter*, *Morganella*, *Serratia*, and *Citrobacter freundii*; intrinsic ampicillin resistance was excluded for *Klebsiella* species (chromosomal SHV-1/LEN-1); standard-method aminoglycoside resistance was excluded for *Enterococcus* species (reflecting intrinsic low-level resistance rather than high-level synergy resistance); and nitrofurantoin was excluded for all organisms as it does not appear in any Magiorakos category table. For *Enterococcus*, amoxicillin-clavulanate susceptibility was accepted as equivalent to ampicillin for the penicillin category, given the absence of β-lactamase production in this genus. Only isolates tested against at least three applicable organism-specific categories were included in the MDR denominator. Polymicrobial culture entries were excluded from the MDR evaluation because susceptibility results could not be attributed to individual organisms.

### 4.8. Outcomes

The primary outcomes were (i) the distribution of causative uropathogens isolated from culture-proven UTI episodes in pediatric patients with congenital urogenital anomalies, and (ii) the proportion of non-susceptible isolates per antibiotic and per antibiotic class. Secondary outcomes were (iii) the prevalence of multidrug resistance per the Magiorakos consensus criteria with intrinsic resistance excluded; (iv) candidate predictors of multidrug resistance at the episode level estimated by GEE logistic regression; and (v) descriptive comparison of antimicrobial resistance proportions between patients with single versus recurrent infection episodes. All outcomes were defined operationally using prespecified analysis criteria; exploratory subgroup signals are reported as such throughout the manuscript.

### 4.9. Statistical Analysis

Descriptive statistics were calculated for all study variables. Distribution of continuous variables was assessed using the Shapiro–Wilk test; right-skewed variables (notably age at first UTI and infection episode count per patient, both Shapiro–Wilk *p* < 0.001) are reported as median [interquartile range, IQR] alongside mean ± standard deviation (SD) to allow comparability with prior pediatric UTI literature, while categorical variables are expressed as frequencies and percentages. The principal inferential analyses (logistic generalized estimating equations and Cochran–Armitage trend testing) do not require normality of predictors; the resistance and MDR outcomes are categorical and were modeled accordingly. Antimicrobial resistance rates were calculated as the proportion of non-susceptible isolates (categorical ‘I’ or ‘R’ as reported in the laboratory information system, combined per the Magiorakos et al. consensus framework for acquired antimicrobial resistance [25]) among those tested for each antibiotic or antibiotic class. Isolates reported as intermediate (‘I’) represented a very small fraction of all susceptibility tests (23/6233, 0.37%); a sensitivity analysis reclassifying ‘I’ as susceptible produced essentially identical estimates given the rarity of intermediate values, and we report the Magiorakos-aligned non-susceptibility convention as the primary analysis throughout the manuscript and tables. The 95% confidence intervals for proportions were calculated using the Wilson score method, which provides reliable coverage for proportions near 0 or 1 and for smaller sample sizes [26]. To evaluate candidate predictors of multidrug resistance at the episode level, we fitted a multivariable logistic regression model (MDR status as outcome) with age group (infant/toddler/child/adolescent; reference: infant), sex (reference: female), CAKUT phenotype (hydronephrosis [reference], hypospadias, posterior urethral valves [PUVs], vesicoureteral reflux [VUR], other [*n* = 15 episodes, retained for completeness but interpreted cautiously given small cell size]), and recurrent infection status (≥2 episodes) were used as predictors. Because patients contributed variable numbers of episodes (1–14), the model was fitted using generalized estimating equations (GEEs) with an exchangeable working correlation structure, clustered on patient identifier (MRN), to account for within-patient correlation of episodes. Adjusted odds ratios (aORs) with 95% Wald confidence intervals are reported. Of the 168 patients in the cohort, 156 contributed at least one episode tested against ≥3 organism-specific Magiorakos categories and were included in the multidrug resistance multivariable analyses; the remaining 12 patients had episodes tested against fewer categories and could not be classified as MDR per the consensus definition. As a sensitivity analysis, the multivariable model was refitted with available structured procedural exposure proxies extracted from the patient-level dataset: any documented surgical procedure (binary, derived from the structured surgery field), documented stent/catheter/vesicostomy exposure (binary), and total infection episode count (continuous). The structured surgery field in the patient-level dataset captures the most recent procedure type per patient; a documented surgery event count per patient (Num_Surgeries) was approximated by deduplicating surgery/surgery date/time/surgical case specialty tuples in the row-level surgical records, but this remains a structured data proxy and does not capture surgery timing relative to the UTI episode, catheter or stent duration, continuous antibiotic prophylaxis regimen, hospital admission count, length of stay, or prior antibiotic prescription history. Where the surgery field was empty, the value was coded as ‘no documented procedure’ for the primary extended sensitivity model. As an additional documented procedure subgroup sensitivity analysis, we refitted the model restricted to the 106 patients (252 episodes) with a non-empty surgery field; this is interpreted as a secondary subgroup analysis (answering whether the hypospadias signal is observed within the documented surgery subgroup) rather than a complete-case correction, because we cannot establish from the available data whether empty surgery values represent missing data or true absence of a documented procedure. As a further sensitivity, we additionally derived a count of distinct documented surgical procedures per patient (Num_Surgeries) from the row-level surgical records by counting unique (surgery, surgery date/time, surgical case specialty) tuples per MRN, and refitted the extended model with Num_Surgeries added as a continuous covariate. Because the hypospadias/epispadias category included three records requiring clinical classification review, we performed an additional classification sensitivity analysis refitting both the primary model and the extended Model C′ on the remaining 153 patients (362 episodes). Annual MDR proportions across 2015–2025 were evaluated descriptively and formally assessed using the Cochran–Armitage linear-by-linear chi-square trend test. As a sensitivity analysis to address potential pseudo-replication from patients contributing multiple episodes, key resistance rates and MDR estimates were also computed using only the first isolate per patient: class-level resistance proportions used the full first isolate cohort (*n* = 168), whereas the first isolate MDR sensitivity analysis was restricted to isolates with at least three applicable Magiorakos categories tested (*n* = 156), and both analyses were compared descriptively with the primary episode-level analysis. All statistical analyses were performed using Python version 3.9 with the pandas, statsmodels, and scipy libraries.

## 5. Conclusions

In this 11-year single-center cohort, pediatric patients with congenital urogenital anomalies had high uropathogen non-susceptibility to commonly used first-line agents, including TMP-SMX, penicillin-class agents, and cephalosporins. Approximately one-third of evaluable isolates met organism-specific MDR criteria, and the organism distribution differed from typical community pediatric UTI patterns, with lower *E. coli* predominance and higher proportions of *Klebsiella* and *Pseudomonas* species.

Within the limits of retrospective laboratory data without linked clinical outcomes, carbapenems and aminoglycosides retained microbiological activity, whereas TMP-SMX and ampicillin appeared poorly suited for empiric monotherapy in this tertiary referral setting. These findings should be used as institution-level surveillance data rather than generalizable treatment guidance. Larger multicenter studies linking resistance patterns to clinical outcomes, procedural exposure, prophylaxis, and molecular resistance mechanisms are needed.

## Figures and Tables

**Table 1:** Demographics and clinical characteristics of 168 pediatric patients with congenital urogenital anomalies and UTI (2015–2025).

Characteristic	Value
Total patients	168
Male	121 (72.0%)
Female	47 (28.0%)
Age (mean ± SD)	4.1 ± 4.5
Infant (<1 y)	47 (28.0%)
Toddler (1–5 y)	71 (42.3%)
Child (6–12 y)	41 (24.4%)
Adolescent (13–17 y)	9 (5.4%)
Culture-positive UTI episodes	411

**Table 2:** Organism-specific antimicrobial resistance patterns.

Organism	n Isolates	TMP-SMX	Pen	Nitro	Ceph	FQ	AG	Carb
*Escherichia coli*	150	64.3	30.4	5.1	29.1	30.9	8.6	0.2
*Pseudomonas aeruginosa*	33	IR ^†^	68.8	IR ^†^	52.7	13.1	6.2	23.2
*Klebsiella pneumoniae*	96	47.7	37.2	22.4	32.0	10.2	5.7	0.7
*Acinetobacter baumannii*	11	18.2	30.8	IR ^†^	44.0	0.0	0.0	23.5
*Enterobacter cloacae*	15	46.7	61.5	45.5	39.6	7.4	13.6	0.0

Values represent the percentage of non-susceptible isolates (resistant + intermediate). TMP-SMX, trimethoprim-sulfamethoxazole; Pen, penicillins; Nitro, nitrofurantoin; Ceph, cephalosporins; FQ, fluoroquinolones; AG, aminoglycosides; Carb, carbapenems. ^†^ IR: intrinsic resistance. *P. aeruginosa* is intrinsically non-susceptible to both nitrofurantoin and trimethoprim-sulfamethoxazole (TMP-SMX), and *Acinetobacter baumannii* to nitrofurantoin, per CLSI intrinsic resistance guidance; these agents are not routinely interpreted against these organisms. Corresponding cells are marked IR and were excluded from pooled class-level non-susceptibility calculations.

**Table 3:** Multidrug resistance (MDR) Rates by Organism.

Organism	n Tested	n MDR	% MDR (95% CI)
*Klebsiella oxytoca*	7	4	57.1 (25.0–84.2)
*Proteus mirabilis*	12	6	50.0 (25.4–74.6)
*Escherichia coli*	147	70	47.6 (39.7–55.7)
*Morganella morganii*	5	2	40.0 (11.8–76.9)
*Klebsiella pneumoniae*	94	36	38.3 (29.1–48.4)
*Enterobacter cloacae*	15	4	26.7 (10.9–52.0)
*Serratia marcescens*	6	1	16.7 (3.0–56.4)
*Pseudomonas aeruginosa*	33	2	6.1 (1.7–19.6)
*Enterococcus faecalis*	16	0	0.0 (0.0–19.4)
*Acinetobacter baumannii*	11	0	0.0 (0.0–25.9)
*Pseudomonas fluorescens*	5	0	0.0 (0.0–43.4)
Other (<5 isolates)	18	6	33.3 (16.3–56.3)
Total	369	131	35.5 (30.8–40.5)

Per-organism rows are shown for organisms with ≥5 MDR-evaluable isolates (*n* = 351 of the 369 total evaluable isolates); 18 episodes from less common organisms (contributing 6 MDR cases) are included in the Total row but not shown as individual rows. Abbreviations: MDR, multidrug resistance per the Magiorakos consensus criteria; CI, confidence interval (Wilson score).

**Table 4:** Multivariable logistic regression for predictors of multidrug resistance.

Predictor	Adjusted OR	95% CI	*p*-Value
Age group (ref: Infant < 1 y)			
Toddler	1.17	0.66–2.09	0.590
Child	0.56	0.25–1.25	0.158
Adolescent	0.71	0.23–2.19	0.546
Sex (ref: Female)			
Male sex	0.34	0.16–0.73	**0.006**
CAKUT phenotype (ref: Hydronephrosis)			
Hypospadias	2.07	1.03–4.14	**0.040**
PUV	1.66	0.79–3.49	0.178
VUR	1.82	0.75–4.40	0.183
Other	0.48	0.09–2.43	0.373
Infection history			
Recurrent infections (≥2 episodes)	1.12	0.62–2.03	0.703

The hypospadias point estimate is sensitive to model specification; see Supplementary Table S6 for the four-model sensitivity analysis (Model A primary aOR 2.07, Model B extended 2.03, Model B-DocSurg subgroup 1.71, Model C′ procedure count 2.08). OR, odds ratio; CI, confidence interval; PUVs, posterior urethral valves; VUR, vesicoureteral reflux. Model: generalized estimating equation (GEE) logistic regression with exchangeable working correlation, clustered on patient identifier to account for multiple episodes per patient. *n* = 369 evaluable isolates across 156 patients (isolates with at least three Magiorakos categories tested). Bolded values indicate *p* < 0.05.

## Data Availability

The datasets generated and analyzed during the current study are not publicly available due to patient privacy considerations and institutional data governance policies but are available from the corresponding author on reasonable request subject to appropriate data sharing agreements.

## Supplementary Materials

The following supporting information can be downloaded at: https://www.mdpi.com/article/10.3390/antibiotics15050506/s1. Table S1. Distribution of Causative Organisms; Table S2. Organism Distribution by Age Group; Table S3. Antimicrobial Non-Susceptibility Rates by Antibiotic Class; Table S4. Antimicrobial Non-Susceptibility by Recurrence Status; Table S5. Individual Antibiotic Susceptibility Rates; Table S6. Extended Multivariable Logistic Regression (Sensitivity Models) for Predictors of Multidrug Resistance; Table S7. Age-Stratified MDR Prevalence and Class-Level Non-Susceptibility; Figure S1. Distribution of Uropathogens.
