# Supplementary material for: Antimicrobial Resistance in Pediatric UTIs with Congenital Urogenital Anomalies: An 11-Year Saudi Retrospective Study
Source: Antibiotics (Basel). 2026 May 18;15(5):506. doi: 10.3390/antibiotics15050506 (PMC13203643; doi:10.3390/antibiotics15050506)
Supplement: Supplementary file 1 [file antibiotics-15-00506-s001.zip › Supplementary_Table_S6_Extended_GEE.pdf]

Supplementary Table S6. Extended Multivariable Logistic Regression (Sensitivity Models) for Predictors of Multidrug Resistance.

Sensitivity analysis: four GEE logistic-regression models for MDR (exchangeable working correlation, clustered on MRN). Model A (primary, identical to Table 4): n = 369 episodes / 156 patients; adjusted for age group, sex, anomaly phenotype, and recurrence. Model B: A plus three structured procedural-exposure proxies (Has_Surgery, stent / catheter / vesicostomy, total episode count); same n. Model B-DocSurg: B restricted to 106 patients with non-empty Surgery field (n = 252 episodes). Model C': B with Num_Surgeries (count of distinct surgical procedures per patient, range 0-13) replacing the binary Has_Surgery to avoid collinearity. Full methodological detail in Methods §2.9; interpretation in Discussion §4.

Term	Model A (primary)<br>aOR (95% CI), p	Model B (extended)<br>aOR (95% CI), p	Model B-DocSurg (subgroup)<br>aOR (95% CI), p	Model C' (Num_Surgeries replaces Has_Surgery)<br>aOR (95% CI), p
**Intercept**	0.84 (0.36-1.99), p = 0.696	0.78 (0.31-1.99), p = 0.602	1.25 (0.44-3.54), p = 0.671	0.83 (0.35-1.98), p = 0.677
**AgeGroup = Toddler**	1.17 (0.66-2.09), p = 0.590	1.12 (0.63-2.01), p = 0.693	1.02 (0.55-1.90), p = 0.953	1.12 (0.63-2.00), p = 0.706
**AgeGroup = Child**	0.56 (0.25-1.25), p = 0.158	0.52 (0.22-1.20), p = 0.125	0.41 (0.16-1.03), p = 0.057	0.51 (0.22-1.19), p = 0.119
**AgeGroup = Adolescent**	0.71 (0.23-2.19), p = 0.546	0.64 (0.19-2.09), p = 0.458	0.48 (0.14-1.62), p = 0.238	0.65 (0.20-2.08), p = 0.470
**Sex = Male**	**0.34 (0.16-0.73), p = 0.006**	**0.32 (0.15-0.70), p = 0.004**	**0.27 (0.11-0.68), p = 0.005**	**0.33 (0.15-0.72), p = 0.005**
**Anomaly = Hypospadias**	**2.07 (1.03-4.14), p = 0.040**	2.03 (0.98-4.23), p = 0.058	1.71 (0.80-3.67), p = 0.165	**2.08 (1.02-4.25), p = 0.045**
**Anomaly = PUV**	1.66 (0.79-3.49), p = 0.179	1.61 (0.76-3.42), p = 0.213	2.20 (0.99-4.92), p = 0.054	1.63 (0.77-3.46), p = 0.202
**Anomaly = VUR**	1.82 (0.75-4.40), p = 0.183	1.79 (0.75-4.26), p = 0.189	1.41 (0.51-3.86), p = 0.508	1.83 (0.77-4.32), p = 0.169
**Anomaly = Other**	0.48 (0.09-2.43), p = 0.373	0.52 (0.10-2.65), p = 0.433	1.32 (0.28-6.21), p = 0.727	0.50 (0.10-2.55), p = 0.402
**Recurrent**	1.12 (0.62-2.03), p = 0.703	1.04 (0.53-2.03), p = 0.920	1.25 (0.57-2.74), p = 0.575	1.02 (0.52-2.01), p = 0.952
**Has_Surgery_Documented**	—	1.21 (0.67-2.20), p = 0.524	—	—
**Has_Stent_or_Catheter**	—	0.74 (0.38-1.47), p = 0.397	1.12 (0.59-2.12), p = 0.738	0.69 (0.32-1.48), p = 0.342
**Num_Infection_Episodes**	—	1.03 (0.95-1.10), p = 0.482	0.97 (0.90-1.03), p = 0.319	1.02 (0.95-1.10), p = 0.536
**Num_Surgeries**	—	—	—	1.04 (0.94-1.16), p = 0.425

Abbreviations: aOR = adjusted odds ratio; CI = confidence interval; PUV = posterior urethral valves; VUR = vesicoureteral reflux; GEE = generalized estimating equations; MDR = multidrug resistance.
