# Supplementary material for: Antimicrobial Resistance in Pediatric UTIs with Congenital Urogenital Anomalies: An 11-Year Saudi Retrospective Study
Source: Antibiotics (Basel). 2026 May 18;15(5):506. doi: 10.3390/antibiotics15050506 (PMC13203643; doi:10.3390/antibiotics15050506)
Supplement: Supplementary file 1 [file antibiotics-15-00506-s001.zip › Supplementary_Table_S7_AgeStratified.pdf]

Supplementary Table S7. Age-Stratified MDR Prevalence and Class-Level Non-Susceptibility.

Age-stratified descriptive summary requested by Reviewer 1 point 1.6. Panel A: MDR prevalence by age group within the MDR-evaluable cohort (n = 156 patients, 369 episodes). Panel B: class-level non-susceptibility (intermediate + resistant; I+R) by age group at the test-result level (n = 168 cohort patients). MDR proportions were broadly similar across age groups (29.6%-39.1%); class-level patterns showed no systematic age-dependent shifts. The Adolescent stratum is small (n = 9 patients / 21 episodes); descriptive interpretation only.

Panel A. Multidrug-Resistance (MDR) Rate by Age Group

Age group	Patients (n)	Episodes (n)	MDR (n)	MDR % (95% CI)
**Infant (<1y)**	45	99	36	36.4 (27.6-46.2)
**Toddler (1-5y)**	64	151	59	39.1 (31.7-47.0)
**Child (6-12y)**	38	98	29	29.6 (21.5-39.3)
**Adolescent (13-17y)**	9	21	7	33.3 (17.2-54.6)
**Total**	**156**	**369**	**131**	**35.5 (30.8-40.5)**

Panel B. Class-Level Non-Susceptibility by Age Group (% non-susceptible (95% CI); n / N tested). See Supplementary Table S5 for individual-drug I and R breakdown.

Antibiotic class	Infant (<1y)	Toddler (1-5y)	Child (6-12y)	Adolescent (13-17y)	Total
**TMP-SMX**	62.7 (52-72); 52/83	62.4 (54-70); 93/149	37.4 (28-48); 34/91	35.0 (18-57); 7/20	54.2 (49-59); 186/343
**Penicillins**	41.5 (36-48); 103/248	38.8 (34-44); 167/430	39.8 (34-46); 109/274	39.6 (28-53); 21/53	39.8 (37-43); 400/1005
**Nitrofurantoin**	20.7 (14-30); 18/87	30.9 (24-39); 46/149	34.0 (25-44); 32/94	21.1 (9-43); 4/19	28.7 (24-34); 100/349
**Cephalosporins**	29.5 (25-35); 89/302	39.1 (35-43); 197/504	32.2 (27-37); 106/329	25.0 (16-36); 17/68	34.0 (31-37); 409/1203
**Fluoroquinolones**	20.7 (15-27); 38/184	19.4 (15-24); 60/309	18.7 (14-25); 35/187	7.7 (3-20); 3/39	18.9 (16-22); 136/719
**Aminoglycosides**	15.6 (12-20); 40/257	7.6 (5-10); 34/447	6.2 (4-10); 17/276	7.0 (3-17); 4/57	9.2 (8-11); 95/1037
**Carbapenems**	4.0 (2-7); 10/250	1.6 (1-3); 7/434	2.8 (1-6); 7/251	8.9 (4-19); 5/56	2.9 (2-4); 29/991

Abbreviations: MDR = multidrug resistance per Magiorakos consensus criteria; CI = confidence interval (Wilson); n_NS = number non-susceptible (intermediate + resistant).
