# Supplementary material for: Antimicrobial Resistance in Pediatric UTIs with Congenital Urogenital Anomalies: An 11-Year Saudi Retrospective Study
Source: Antibiotics (Basel). 2026 May 18;15(5):506. doi: 10.3390/antibiotics15050506 (PMC13203643; doi:10.3390/antibiotics15050506)

SUPPLEMENTARY MATERIALS

Antimicrobial Resistance in Pediatric UTIs with Congenital Urogenital Anomalies: An 11-Year Saudi Retrospective Study

Contents:

1. Table S1. Distribution of Causative Organisms
2. Table S2. Organism Distribution by Age Group
3. Table S3. Antimicrobial Non-Susceptibility Rates by Antibiotic Class
4. Table S4. Antimicrobial Non-Susceptibility by Recurrence Status
5. Table S5. Individual Antibiotic Susceptibility Rates
6. Figure S1. Distribution of Uropathogens (moved from main manuscript)

SUPPLEMENTARY TABLES

Table S1. Distribution of Causative Organisms

Organism	n	%
Escherichia coli	150	37.2
Klebsiella pneumoniae	96	23.8
Pseudomonas aeruginosa	33	8.2
Enterococcus faecalis	22	5.5
Enterobacter cloacae	15	3.7
Proteus mirabilis	12	3.0
Acinetobacter baumannii	11	2.7
Klebsiella oxytoca	7	1.7
Serratia marcescens	6	1.5
Morganella morganii	5	1.2
Pseudomonas fluorescens	5	1.2
Citrobacter freundii	5	1.2
Other	36	8.9
Total	403	100.0

Distribution of uropathogens isolated from 403 mono-organism infection episodes (n = 403; 8 polymicrobial cultures, 1.9%, excluded from organism attribution; see Methods §2.2). Organisms with <5 isolates are grouped as "Other".

Table S2. Organism Distribution by Age Group

Age Group	Total Isolates	*Escherichia coli*	*Klebsiella pneumoniae*	*Pseudomonas aeruginosa*	*Enterococcus faecalis*	*Enterobacter cloacae*
Infant (<1y)	103	42 (40.8%)	25 (24.3%)	10 (9.7%)	10 (9.7%)	3 (2.9%)
Toddler (1-5y)	171	61 (35.7%)	36 (21.1%)	10 (5.8%)	9 (5.3%)	11 (6.4%)
Child (6-12y)	114	40 (35.1%)	25 (21.9%)	13 (11.4%)	1 (0.9%)	1 (0.9%)
Adolescent (13-17y)	23	7 (30.4%)	10 (43.5%)	0 (0.0%)	2 (8.7%)	0 (0.0%)

Distribution of the five most common organisms across age groups. Values shown as n (%). Age categories: Infant (<1 year), Toddler (1-5 years), Child (6-12 years), Adolescent (13-17 years).

Table S3. Antimicrobial Non-Susceptibility Rates by Antibiotic Class

Antibiotic Class	n tested	n non-susceptible	% non-susceptible	95% CI
TMP-SMX	343	186	54.2	48.9-59.4
Penicillins	1005	400	39.8	36.8-42.9
Cephalosporins	1203	409	34.0	31.4-36.7
Nitrofurantoin	349	100	28.7	24.2-33.6
Fluoroquinolones	719	136	18.9	16.2-21.9
Aminoglycosides	1037	95	9.2	7.6-11.1
Carbapenems	991	29	2.9	2.0-4.2

Overall antimicrobial non-susceptibility rates (I+R combined per Magiorakos consensus) by antibiotic class. 95% confidence intervals calculated using Wilson score method. Classes include: TMP-SMX (trimethoprim-sulfamethoxazole), Penicillins (ampicillin, amoxicillin-clavulanate, piperacillin-tazobactam), Cephalosporins (ceftazidime, cefepime, ceftriaxone, cefotaxime, cefazolin), Nitrofurantoin, Fluoroquinolones (ciprofloxacin, levofloxacin), Aminoglycosides (gentamicin, amikacin, tobramycin), Carbapenems (meropenem, imipenem, ertapenem).

Table S4. Antimicrobial Non-Susceptibility by Recurrence Status

Recurrence Status	n isolates	TMP-SMX	Penicillins	Nitrofurantoin	Cephalosporins	Fluoroquinolones	Aminoglycosides	Carbapenems
Single Episode	79	48.6%	30.7%	13.9%	28.9%	8.7%	5.4%	1.5%
Recurrent (>=2)	331	55.8%	41.6%	31.0%	34.9%	21.1%	9.8%	3.3%

Comparison of antimicrobial non-susceptibility rates between patients with single episode (n=79 isolates) versus recurrent infections (≥2 episodes, n=331 isolates). Recurrent infections showed numerically higher non-susceptibility rates across all antibiotic classes.

Table S5. Individual Antibiotic Susceptibility Rates

Antibiotic	Class	n tested	Susceptible n (%)	Intermediate n (%)	Resistant n (%)
Gent	Aminoglycosides	366	306 (83.6%)	1 (0.3%)	59 (16.1%)
Tobra	Aminoglycosides	327	295 (90.2%)	1 (0.3%)	31 (9.5%)
Amik	Aminoglycosides	344	341 (99.1%)	0 (0.0%)	3 (0.9%)
Erta	Carbapenems	309	291 (94.2%)	0 (0.0%)	18 (5.8%)
Imi	Carbapenems	343	335 (97.7%)	0 (0.0%)	8 (2.3%)
Mero	Carbapenems	339	336 (99.1%)	0 (0.0%)	3 (0.9%)
Cefaz	Cephalosporins	331	122 (36.9%)	1 (0.3%)	208 (62.8%)
Cefo	Cephalosporins	274	175 (63.9%)	1 (0.4%)	98 (35.8%)
Cefep	Cephalosporins	336	277 (82.4%)	1 (0.3%)	58 (17.3%)
Ceftaz	Cephalosporins	256	215 (84.0%)	0 (0.0%)	41 (16.0%)
Cipro	Fluoroquinolones	361	284 (78.7%)	1 (0.3%)	76 (21.1%)
Levo	Fluoroquinolones	358	299 (83.5%)	2 (0.6%)	57 (15.9%)
Nitro	Nitrofurantoin	349	249 (71.3%)	4 (1.1%)	96 (27.5%)
Azt	Other	268	214 (79.9%)	1 (0.4%)	53 (19.8%)
Col	Other	318	287 (90.3%)	6 (1.9%)	25 (7.9%)
Amp	Penicillins	351	58 (16.5%)	0 (0.0%)	293 (83.5%)
Amox/Cla	Penicillins	327	229 (70.0%)	2 (0.6%)	96 (29.4%)
Pip/Taz	Penicillins	327	318 (97.2%)	2 (0.6%)	7 (2.1%)
SXT	TMP-SMX	343	157 (45.8%)	0 (0.0%)	186 (54.2%)

Detailed susceptibility results for individual antibiotics. S = Susceptible, I = Intermediate, R = Resistant. Only antibiotics with ≥10 tests are shown.

SUPPLEMENTARY FIGURES

Figure S1. Distribution of Uropathogens

Figure S1. Distribution of uropathogens isolated from the 403 mono-organism culture-proven urinary tract infection episodes in 168 pediatric patients with congenital urogenital anomalies treated at King Khalid University Hospital, Riyadh, between 2015 and 2025 (after exclusion of 8 polymicrobial cultures, 1.9%, see Methods §2.2). Numerical counts and proportions for each species are reported in Supplementary Table S1.

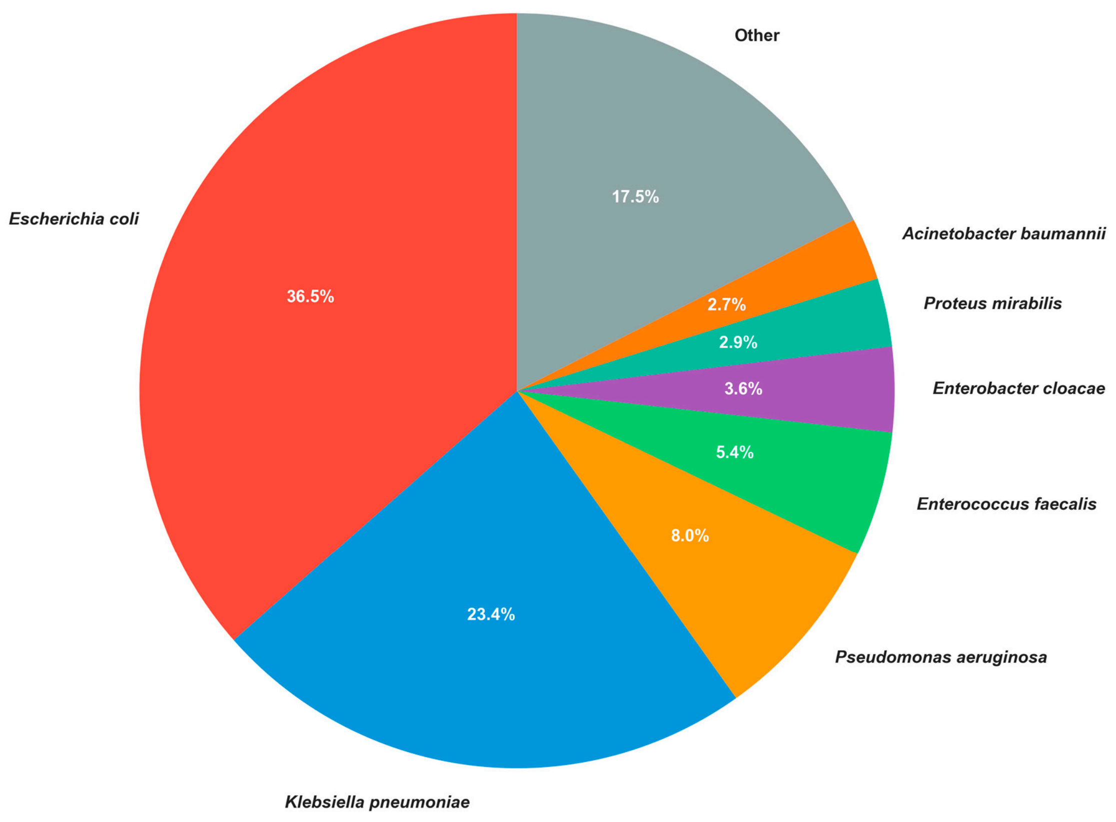

Supplement: Supplementary file 1 [file antibiotics-15-00506-s001.zip › Supplementary_Tables and Figure.pdf]
